# Prediction of Clinical Outcomes in Acute Ischaemic Stroke Patients: A Comparative Study

**DOI:** 10.3389/fneur.2021.663899

**Published:** 2021-05-06

**Authors:** Deepthi Rajashekar, Michael D. Hill, Andrew M. Demchuk, Mayank Goyal, Jens Fiehler, Nils D. Forkert

**Affiliations:** ^1^Biomedical Engineering Graduate Program, University of Calgary, Calgary, AB, Canada; ^2^Depertment of Radiology, Cumming School of Medicine, University of Calgary, Calgary, AB, Canada; ^3^Hotchkiss Brain Institute, University of Calgary, Calgary, AB, Canada; ^4^Department of Clinical Neurosciences, Cumming School of Medicine, University of Calgary, Calgary, AB, Canada; ^5^Department of Medicine, Cumming School of Medicine, University of Calgary, Calgary, AB, Canada; ^6^Department of Community Health Sciences, University of Calgary, Calgary, AB, Canada; ^7^Department of Diagnostic and Interventional Neuroradiology, University Medical Center Hamburg-Eppendorf, Hamburg, Germany; ^8^Alberta Children's Hospital Research Institute, University of Calgary, Calgary, AB, Canada

**Keywords:** support vector machine, lesion symptom mapping, NIHSS (National Institue of Health Stroke Scale), nested regression, ischemic stroke

## Abstract

**Background:** Clinical stroke rehabilitation decision making relies on multi-modal data, including imaging and other clinical assessments. However, most previously described methods for predicting long-term stroke outcomes do not make use of the full multi-modal data available. The aim of this work was to develop and evaluate the benefit of nested regression models that utilise clinical assessments as well as image-based biomarkers to model 30-day NIHSS.

**Method:** 221 subjects were pooled from two prospective trials with follow-up MRI or CT scans, and NIHSS assessed at baseline, as well as 48-hours and 30 days after symptom onset. Three prediction models for 30-day NIHSS were developed using a support vector regression model: one clinical model based on modifiable and non-modifiable risk factors (M_CLINICAL_) and two nested regression models that aggregate clinical and image-based features that differed with respect to the method used for selection of important brain regions for the modelling task. The first model used the widely accepted RreliefF (M_RELIEF_) machine learning method for this purpose, while the second model employed a lesion-symptom mapping technique (M_LSM_) often used in neuroscience to investigate structure-function relationships and identify eloquent regions in the brain.

**Results:** The two nested models achieved a similar performance while considerably outperforming the clinical model. However, M_RELIEF_ required fewer brain regions and achieved a lower mean absolute error than M_LSM_ while being less computationally expensive.

**Conclusion:** Aggregating clinical and imaging information leads to considerably better outcome prediction models. While lesion-symptom mapping is a useful tool to investigate structure-function relationships of the brain, it does not lead to better outcome predictions compared to a simple data-driven feature selection approach, which is less computationally expensive and easier to implement.

## Introduction

The prognosis of clinical and functional outcome in acute ischemic stroke patients is typically made based on multi-modal information such as demographic, clinical, laboratory, and radiological data. Theoretically, machine learning models can identify patterns in high-dimensional data that can be used to make data-driven and reproducible stroke outcome predictions in new patients and support patient management. However, despite the ability to integrate multimodal information, recent machine learning models have mostly utilized clinical data or image-based biomarkers alone (1) to predict stroke outcome. So far, the benefit of using true multi-modal data for stroke outcome prediction has not been investigated comprehensively. One of the few multi-modal predictive models of stroke outcome is described by Brugnara et al. (2). However, clinical assessments at various timepoints are used as input features without addressing the issue of feature collinearity. Furthermore, previous studies often predict the stroke outcome in a binary classification scheme (good vs. bad), which ignores the incremental, yet relevant non-linear differences in stroke severity scores.

Integration of image-based biomarkers for stroke outcome prediction is more complex than using other clinical assessments (in most cases), but has the potential to add considerable predictive power. A key aspect to consider within this context is the selection of regions-of-interest (ROIs) in the brain that are critically associated with the clinical deficit of interest since non-informative and redundant feature can downgrade the prediction accuracy considerably (3). Lesion-symptom mapping (LSM) (4) is able to identify brain regions that are important for a clinical outcome score of interest but has been used rarely for selection of brain regions for stroke outcome prediction (5). The more common ROI selection approach is to use classical feature selection methods during the training process. However, these two general approaches have never been compared to date with respect to stroke outcome prediction.

The aim of this work is to compare different setups of nested machine learning models using clinical information only and a combination of clinical and radiological features selected using lesion-symptom mapping and classical feature selection methods to predict the 30-days NIH stroke scale (NIHSS).

## Methods

### Data

The datasets used in this study were pooled from the ESCAPE (6) and iKNOW (7) trials. Patients with remote hemorrhages, bilateral lesions, and severe white matter hyperintensities were excluded from this secondary analysis, and only patients with a follow-up MRI or CT scan (18-hours to one week from baseline) with complete clinical information (obtained after stroke and upto 6-hours post randomization) were included, leading to a final sample of 221 patients. The clinical outcome of interest in this study is the NIHSS assessed at 30 days after stroke symptom onset. The patient characteristics are summarized in Table 1. The measurable clinical and laboratory features used in the nested regression model include age, sex, modifiable and non-modifiable risk factors suggested in the evidence-based review of stroke rehabilitation (8). These include blood pressure, glucose, hematocrit, hypertension, diabetes, smoking status, hyperlipidemia, and atrial fibrillation (see Supplementary Table 1). Additionally, the baseline NIHSS score (pre-treatment) was also included as part of the clinical data to model stroke outcome (5, 9).

**Table 1:** Characteristics of patients pooled (*N* = 221) from the ESCAPE^6^ and iKNOW^7^ datasets.

**Variable**	**ESCAPE (*N* = 143)**	**iKNOW (*N* = 78)**	**Dataset (*N* = 221)**
Median Age (IQR)	68 (19.5)	70.5 (15)	69 (19)
Sex—Females	75	31	106
Treatment—Alteplase	66	46	112
Median Onset to randomization time (IQR)	160 min (149)	126.5 min (118.05)	152 min (137)
Median Baseline NIHSS (IQR)	16 (7)	12 (10)	15 (8)

All lesions were manually delineated by an expert observer using the ITKSNAP tool. Each image sequence was skull stripped and non-linearly registered to the common FLAIR-NCCT (10) atlas of the elderly using the ANTs toolkit. The grey matter (GM) and white matter (WM) parcellations from the probabilistic BNA atlas (11) and the JHU atlas (12), respectively, were fused and transformed to the FLAIR-NCCT atlas. All image-based features were computed in the FLAIR-NCCT atlas space.

### Model Design

Nested regression models were developed to predict the 30-days NIHSS outcome based on clinical data and image-based biomarkers. Here, the first model predicts the 48-hours NIHSS using imaging features alone whereas the result of this model is then used together with clinical features to predict the 30-days NIHSS.

$$\begin{array}{l} \;NIHSS_{30-days}\sim \;(Features_{Clinical}\\ +\left(NIHSS_{48-hours}\;\sim \;Features_{Imaging}\right)) \end{array}$$

For both models, epsilon-regression was used implemented using in a radial kernel support vector regression (SVR) framework. Using follow-up imaging acquired between 18-hours and 5-days from symptom onset to identify regions-of-interest (ROIs) that maximally correlate with a long-term assessment might introduce confounding effects and bias the results. Therefore, the ROIs included in the predictive models were identified with respect to the 48-hours NIHSS to ensure that the identified structure-function relationships are related to the primary stroke-induced deficits alone. This also ensures that the identified ROIs are not selected because of post-secondary comorbidities (not directly related to the primary stroke) developed either in-hospital or post-discharge. The two approaches for ROI selection are: (i) the LSM method using Brunner-Munzel test (13) and (ii) a widely accepted machine learning-based feature selection method that accounts for collinearity known as RreliefF (14). The LSM method was implemented using the LESYMAP package (15) using the default parameters employing a p-value threshold at 0.05, discarding voxels not injured in at least 10% of the sample data, and using false discovery rate (FDR, the rate of Type 1 errors) to correct for multiple comparisons. For ease of comparison, brain regions that were not affected in at least 10% of the sample data were also removed prior to the RreliefF feature selection. The RreliefF feature selector was also employed using default parameters from the Fselector package (16) with the sample size of 10 and a neighbor count set to five. The result of the LSM is a statistical map of clusters of significant voxels that survive the FDR correction with non-zero voxel weights. Regions in the BNA-JHU parcellation that were assigned non-zero voxel weights by the LSM analysis were included as ROIs in the proposed regression analyses.

For each brain region identified by LSM as being important in the training set, the relative lesion overlap was computed and used as image-based features. Moreover, in case of WM tracts, the cross-sectional width of the tract spared after the lesion was also calculated and used as additional features (17). Therefore, the final set of image-derived input features used in this study include GM overlap, WM overlap, and WM tract integrity for all the selected ROIs.

For RreliefF feature selection, the lesion overlap (GM and WM) and tract integrity (only WM) was calculated for each atlas region and used for feature selection based on the training set.

### Model Evaluation

For the sake of being able to compare brain regions selected for stroke outcome prediction qualitatively between the two models, the data was randomly split into completely independent training and test sets. This resulted in only one set of features selected for each method, which greatly enhances the interpretability and comparison of the models. Therefore, the entire dataset was partitioned into two mutually exclusive subsets for model training (80%) and testing (20%) using a stratified split that preserves the representation of stroke severity across both groups. Three models were evaluated in this framework: (i) un-nested SVR model with clinical features alone (M_CLINICAL_) selected using RreliefF; (ii) nested model using clinical and imaging data with RreliefF as feature selector (M_RELIEF_); and (iii) nested model using clinical and image data with LSM as feature selector (M_LSM_). The resulting models were compared for predictive performance with respect to the model's mean absolute error (MAE) and coefficient of determination (R^2^).

## Results

The overlap of all individual patient lesions in the atlas space shows that maximum incidence of stroke in this dataset occurs in the brain regions supplied by the middle cerebral artery (see Supplementary Figure 1). The median recovery profile of patients in this database is shown in Supplementary Figure 2.

The model using clinical features only resulted in a rather poor predictive performance (*R*^2^ = 0.13). The optimal prediction results were achieved using age, baseline NIHSS, blood glucose and hematocrit levels, sex, presence of atrial fibrillation, hypertension, and hyperlipidemia, treatment decision (endovascular thrombectomy or tissue plasminogen activator), symptom onset to admission time, and blood pressure as features. However, the iterative feature selection procedure using RreliefF did not select presence of diabetes and smoking status, which are usually considered important predictors. Only the clinical features selected in this model were included in the two nested models to enable a direct comparison.

**Figure 1:**
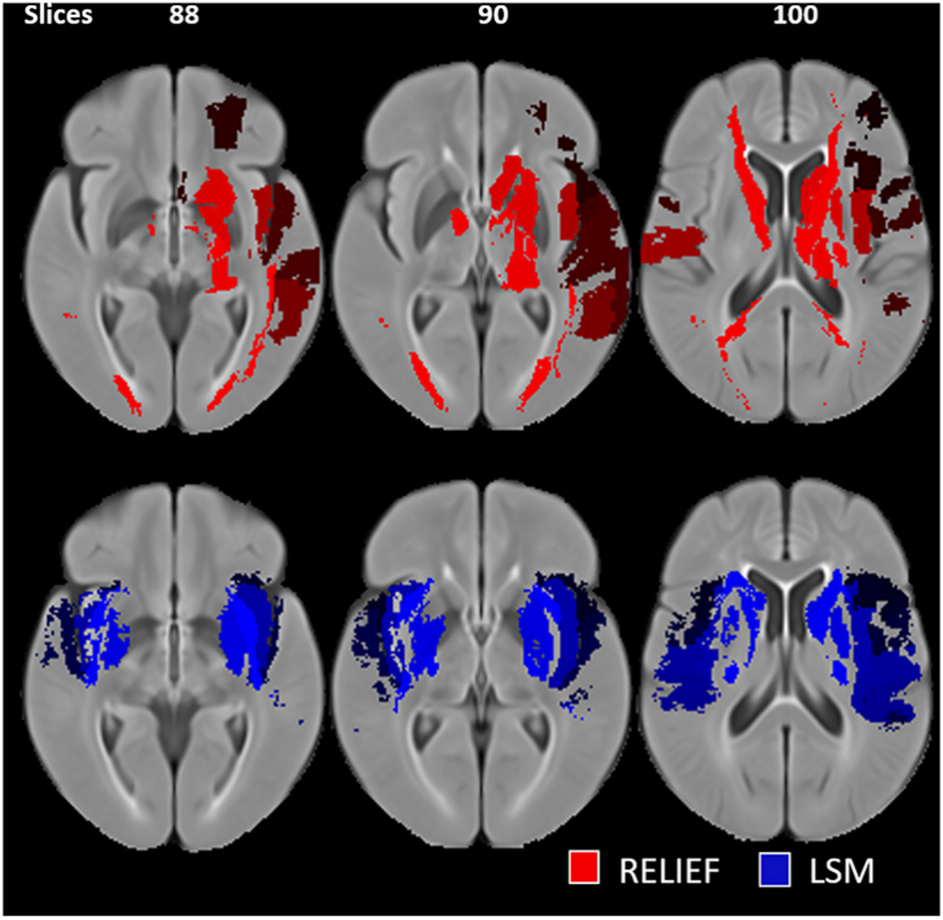
Selected regions of interest (ROIs) for the RreliefF-based (red) and LSM-based (blue) feature selection. The LSM-based ROIs are hemispherically asymmetrical and include regions outside of the subcortical nuclei.

Compared to the simple predictive model using clinical features only, the two nested (M_RELIEF_ and M_LSM_) models performed better and resulted in comparable *R*^2^ and MAEs (see Table 2). No statistically significant MAE differences (*p* > 0.05) were found comparing the two nested models. However, M_RELIEF_ used only 44 ROIs in comparison to the 106 ROIs selected in M_LSM_ (see Figure 1). The plots of the predicted and ground truth scores for both models are shown in Supplementary Figure 3.

**Table 2:** Model performances for each setup.

**Model**	**MAE**	**RMSE**	**R^**2**^**	***p*-value**
M_CLINICAL_	4.33	5.53	0.13	0.0184
M_RELIEF_	3.55	4.34	0.43	1.89e-06
M_LSM_	3.50	4.54	0.40	6.22e-06

*MAE, mean absolute error; RMSE, Root mean squared error; R^2^, coefficient of determination; M_CLINICAL_, model with clinical features alone; M_RELIEF_, ROIs selected by RreliefF and nested with clinical features; M_LSM_, ROIs selected using lesion-symptom mapping and nested with clinical features*.

## Discussion

This study demonstrates that conventional machine learning feature selection methods (M_RELIEF_) can identify important brain regions for stroke outcome prediction as well as the conventional lesion-symptom mapping methods (M_LSM_).

The advantage of the M_RELIEF_ model over the M_LSM_ model are two-fold. First, the M_RELIEF_ model is simpler since it uses <50% of features compared to the M_LSM_ model and results in similar predictive performance. Second, the M_RELIEF_ setup does not require extensive LSM computations to derive structure-function relationships and identify eloquent brain regions. Specifically, despite using a fewer number of regions, the ROIs chosen by the M_RELIEF_ model are largely in the left hemisphere and include regions that correspond to the dominance of left-hemispheric functions assessed by NIHSS.

Importantly, using LSM for ROI selection has additional limitations that the RreliefF feature selection overcomes. First, LSM analyses suffer from low statistical power due to the corrections for multiple comparisons and do not account for violating assumptions of normality in the outcome score. Second, the LSM analysis results in individual voxel weights, which are not really needed to compute region-level inferences of critical brain regions that are associated with a deficit. While LSM is a powerful tool to investigate the neural correlates of stroke induced clinical deficit, its usefulness to select ROIs for stroke outcome prediction tasks seems rather limited. For these reasons, and by applying the Occam's razor principle in model selection, traditional feature selection methods seem to be better suited for future research in stroke outcome prediction.

The proposed framework has a design advantage in comparison to the existing prognostic models of stroke outcome. A recent review on predictive models of stroke outcome (18) reports that: (i) the target outcome of the predictive model is usually a categorized version of functional outcome^1^; (ii) the variables used to model this score include prognostic parameters^2^, stroke risk factors, and baseline stroke severity measured by the NIHSS scale. An obvious limitation is that classification models predicting binarized functional outcome likely ignore the gradation of stroke severity, which is relevant information for stroke prognostication. Furthermore, the functional outcomes, prognostic parameters, and the baseline severity measures may be strongly correlated resulting in inflated classification accuracies. In the proposed work, both of these limitations (loss of relevant information and collinearity) are addressed by employing the nested regression model. For instance, since the 48-hours NIHSS is highly correlated with the 30-day NIHSS, it might bias the regression model. Therefore, having a nested model that utilizes the short-term outcome to derive image-based ROIs that in turn predict the long-term outcomes seems to be a promising way to reduce the affects of collinearity. Furthermore, it is important to note that the results of different studies describing predictive models are not comparable because of different sample sizes, different evaluation methods, different assessment time points, and different imaging time points. For this reason, the predictive model using clinical data only was included in this study as a means of baseline comparison.

One of the limitations of the proposed work is that the findings are population-specific and are likely to change with the stroke cohort used (type of stroke and sample size), choice of parcellation atlas, LSM technique, and/or training scheme employed. This study is also exploratory in the sense that, subject to availability, the clinical descriptors included are a subset of all potential stroke risk factors reported in the literature. The power calculations for using LSM in predictive analysis has not been explored in this study. Additionally, the burden of preprocessing each patient scan for registration, lesion segmentation, and feature computation is extensive. State-of-the-art deep learning methods have the potential to use 3D MRI or CT scans (without lesion definitions) and do not demand handcrafted image-based features and might not even need manual lesion segmentations. Furthermore, the results of this study can be considered a relevant first step toward building a computer-aided prognosis support tool using explainable machine learning methods. However, the predictive accuracy of the models generated in this study need to be further improved using additional datasets and should be evaluated prospectively using a completely independent dataset.

An important recommendation for future work is to model stroke outcomes using ordinal regression models, which can account for the relative ordering between two values in the NIHSS scale. However, ordinal regression models are more complex and typically require the definition of interval thresholds, which can either be derived from the training data or based on domain knowledge. That said, the results described in this paper will generally hold true for ordinal regression models as well. Confirmatory research in this direction may also benefit from investigating the utility of convolutional neural networks without requiring lesion segmentation to predict long-term stroke outcome as an ordinal regression problem.

## Conclusions

In summary, this study shows that combining clinical and imaging data leads to better stroke outcome predictions compared to using clinical data alone. While lesion-symptom mapping is a powerful neuroscience tool to investigate structure-function relationships in stroke patients, these methods do not appear to have an additional benefit for selecting brain regions important for stroke outcome prediction compared to rather simple and data-driven feature selection methods.

## Data Availability Statement

The acquisition of the datasets for the two trials was approved by the respective local ethics board at each site contributing to the two trials. All datasets used in this secondary study were made available after complete anonymization. Requests to access these datasets should be directed to Nils Forkert, nils.forkert@ucalgary.ca.

## Author Contributions

DR conceptualized, conducted experiments, and drafted the paper. MH contributed data, validated the design of experiments, and critically reviewed the paper. JF and MG contributed data, and reviewed the paper. AD contributed data. NF validated the design of experiments, and critically reviewed the paper. All authors contributed to the article and approved the submitted version.

## Conflict of Interest

JF (all unrelated): Research support: EU, BMBF, BMWi, DFG, Acandis, Medtronic, Microvention, Stryker, Consultancy: Acandis, Cerenovus, Medtronic, Microvention, Penumbra, Phenox, Stryker, Stock: Tegus, Executive functions: University Medical Center Hamburg-Eppendorf, Eppdata GmbH. MH reports grants from Covidien (Medronic LLC), during the conduct of the ESCAPE study; personal fees from Merck, non-financial support from Hoffmann-La Roche Canada Ltd, grants from Covidien (Medtronic), grants from Boehringer-Ingleheim, grants from Stryker Inc., grants from Medtronic LLC, grants from NoNO Inc., outside the submitted work; In addition, MH has a patent Systems and Methods for Assisting in Decision-Making and Triaging for Acute Stroke Patients pending to US Patent office Number: 62/086,077 and owns stock in Calgary Scientific Incorporated, a company that focuses on medical imaging software, is a director of the Canadian Federation of Neurological Sciences, a not-for-profit group and has received grant support from Alberta Innovates Health Solutions, CIHR, Heart & Stroke Foundation of Canada, National Institutes of Neurological Disorders and Stroke. The remaining authors declare that the research was conducted in the absence of any commercial or financial relationships that could be construed as a potential conflict of interest.

## Footnotes

^1^Examples include: the modified Ranking Scale (mRS), NIHSS, Barthel Index, etc.

^2^Examples include: Preadmission Comorbidities, Level of Consciousness, Age, and Neurological Deficit (PLAN); Stroke Prognostication Using Age and National Institutes of Health Stroke Scale (SPAN); Totaled Health Risks in Vascular Events (THRIVE), etc.
