# Supplementary material for: Prediction of Clinical Outcomes in Acute Ischaemic Stroke Patients: A Comparative Study
Source: Front Neurol. 2021 May 6;12:663899. doi: 10.3389/fneur.2021.663899 (PMC8134662; doi:10.3389/fneur.2021.663899)

## Supplemental data

| Variable | ESCAPE (N=143) | iKNOW (N=78) | Dataset (N=221) |
| --- | --- | --- | --- |
| Median Systolic blood pressure (IQR) | 140 mm Hg (27) | 140 mm Hg (20) | 140 mm Hg (24) |
| Median Diastolic blood pressure (IQR) | 80 mm Hg (23.5) | 80 mm Hg (17.75) | 80 mm Hg (20) |
| Median Hematocrit (IQR) | 0.4 % (0.05) | 0.4 % (0.13) | 0.4 % (0.08) |
| Median Glucose (IQR) | 6.7 mM (2.05) | 0.74 mM (10.35) | 6.5 mM (3.5) |
| Smoking^*^ | C=34, N=71, P=38 | C=9, N=42, P=27 | C=43, N=113, P=65 |
| Hypertension | Yes=92 | Yes=52 | Yes=144 |
| Hyperlipidemia | Yes=51 | Yes=33 | Yes=84 |
| Diabetes^#^ | T1=17, T2=20, N=106 | T1=5, T2=7, N=66 | T1=22, T2=27, N=172 |
| Atrial fibrillation | Yes=39 | Yes=15 | Yes = 54 |

Table S1: Clinical parameters of patients in the two trials. ^*^C: current, N: never, P: past. ^#^T1: Type 1 diabetes, T2: Type 2 diabetes, N: No diabetes.


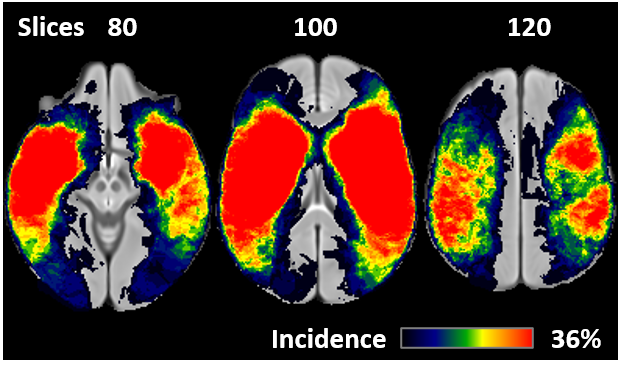


Figure S1: Overlap of all 221 lesions in the MIPLAB atlas space: brain regions that have the maximum stroke incidence are around the subcortical nuclei and insular lobes.


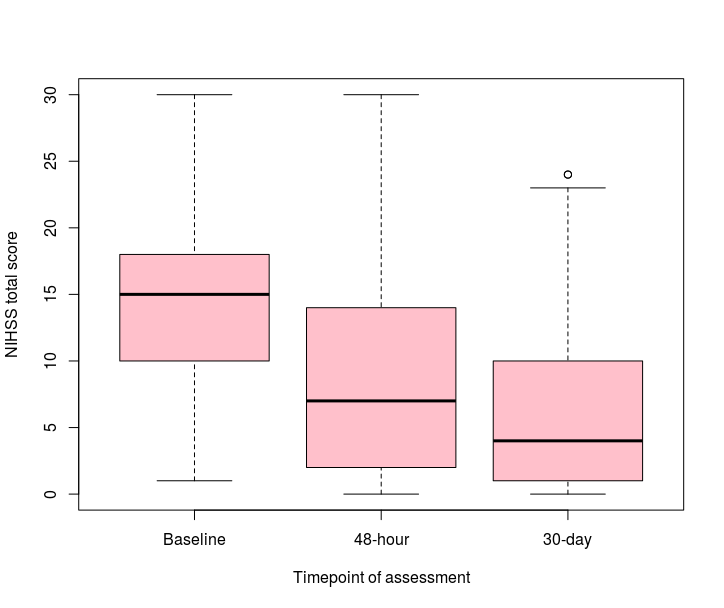


Figure S2: Median NIHSS total scores of the entire dataset (N=221) at three assessment timepoints.

Figure S3: Predicted versus ground truth plot for M_LSM_ and M_RELIEF_ models.


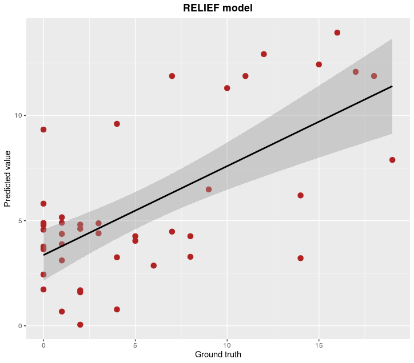

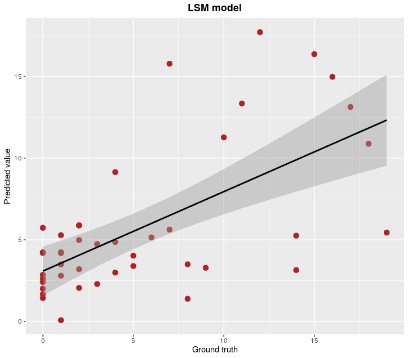

Supplement: Supplementary file 1 [file Data_Sheet_1.docx]
